# Differential wing polyphenism adaptation across life stages under extreme high temperatures in corn leaf aphid

**DOI:** 10.1038/s41598-019-45045-x

**Published:** 2019-06-19

**Authors:** Yu Chen, François J. Verheggen, Dandan Sun, Zhenying Wang, Frederic Francis, KangLai He

**Affiliations:** 1 State Key Laboratory for Biology of Plant Disease and Insect Pests, Institute of Plant Protection, Chinese Academy of Agricultural Science, Beijing, 100193 China; 2 Functional and Evolutionary Entomology, Gembloux Agro-Bio Tech, Liège University, Passage des Déportés, 2, Gembloux, Belgium

**Keywords:** Ecology, Entomology

## Abstract

Polyphenism, a common phenomenon in nature, is an important form of adaptation in a diverse environment. Corn leaf aphid (CLA), *Rhopalosiphum maidis*, (Hemiptera: Aphididae), exhibit wing polyphenism in response to poor habitat quality. In this study, we focused on the effects of crowding and thermal cues on morph determination of CLA. Five developmental stages of aphids (1^st^ to 4^th^ nymphs and maternal adults) with increased population densities, were tested under two kinds of temperature patterns, i.e., A) a constant temperature of 22 °C with 2 h exposure to high temperature in the range of 35 to 39 °C during mid-photophase and B) different constant temperatures in the range of 22–30 °C with 2 h exposure to high temperature of 39 °C during mid-photophase. Crowding was found to directly impact winged induction. The 1^st^ and 2^nd^ nymphs were more sensitive for alate morphs induction under high density. In addition, temperature played a significant role in wing production, with the temperature setting of 26/39 °C in pattern B inducing higher alate morphs and survival than other temperature settings. Therefore, we hypothesize that warmer climate with brief high temperature is more favourable for survival and alate morphs production, but cool weather and transient extreme high temperature (>39 °C) is detrimental for CLA. Our results provide a new perspective on understanding the interactions between changes in extreme high temperatures and insect densities that differentially affect wing polymorphism for further demographic and distribution rates of species across temporal and spatial scales.

## Introduction

Polyphenism is a form of developmental plasticity in which organisms respond to environmental cues by producing adaptive, discrete, alternative phenotypes known as morphs^1^. This phenomenon is common and important as a form of adaptation and a source of variation for natural selection. Particularly, the ambient temperature is the main abiotic factor influencing development and further vital stages in insect life cycles. The impact of extreme high temperature in the context of global warming should than be investigated more precisely considering individual developmental stage for direct effects but also for further adult morph induction. Aphids exhibit polyphenism, as genetically identical individuals can potentially show different phenotypes^2,3^. Compared to the apterous phenotype, the winged aphids have a longer nymphal development period, lower offspring production, and higher longevity^4–7^. Moreover, winged forms are also more resistant to starvation^8^. The morphological and physiological characteristics of winged aphids enable them to survive in harsh conditions, have the chance to disperse and clone to a new environment^9^.

First, a considerable number of studies have addressed the environmental conditions that affect the production of winged individuals in aphids including both biotic (crowding, nutrition, interspecific interactions, natural enemies, alarm pheromone, maternal, etc.) and abiotic (temperature, photoperiod, precipitation, etc.) factors^10–16^. Crowding has been established as a prime factor in the production of winged forms among aphids: higher aphid densities lead to more tactile stimulations between individual aphids, triggering wing induction^17–20^. However, the stage in the life history of the aphid at which crowding has the most influence differs between species^21^. The sensitivity of *Rhopalosiphum insertum* (Walker), *Therioaphis ononidis* (Kalt.), *Brevicoryne brassicae* (L.) and *Myzus persicae* (Sulz.) to crowding seems to be confined almost entirely to the first instar^22–25^. On the other hand, Awram (1968) showed that *M. persicae* may belong to a class of species typified by *Macrosiphum granarium* and *Aphis craccivora* (Koch)^26^, in which prenatal crowding of mothers and postnatal crowding of larvae both influence the production of winged forms^17,27^. However, the sensitive stage of corn leaf aphid (CLA) for winged induction is still unclear.

Second, increased temperature, which is a major response of global change, has a direct impact on the life activities of ectotherms, such as survival, development, fecundity and migration^28–30^. Similar to all ectotherms, aphids have acute sensory capability for detecting temperature variations^31^. There were different opinions in the study of temperature affecting winged aphid induction. Most researches reported that low temperature would induce *M. persicae, L. erysimi, B. brassiae*, *A*. *glycines*, and *Macrosiphoniella sanborni* to become alatae, and high temperature would inhibit wing dimorphism^17,32,33^. However, Diaz and Fereres found that an increase in temperature led to a significant increase in the proportion of alatae lettuce aphid *Nasonovia ribisnigri*^34^. Müller *et al*. pointed out that higher temperature might be expected to influence aphid morph determination indirectly, as associated with the increase in activities and contacts between aphids, which may result in more winged aphids^35^. Interactions between temperature increase and insect density are being studied for the cumulative and critical role on the production of winged aphids.

The corn leaf aphid, *Rhopalosiphum maidis*, (Hemiptera: Aphididae), is an important herbivorous insect pest that feed on corn, barley, millet and many other monocot plants. Similar to most Aphidinae species, CLA can have wings or be wingless, but the roles of particular environmental factors on wing polyphenism have not been examined in this species. The aim of this work was to study the wing polyphenic response both to crowding and thermal cues in CLA, depending on the considered developmental stage under selective pressure. We also considered maternal effect to see whether it is true in our CLA colony. Different temperature patterns were used to identify the thermal environment that induce winged aphids, and the interaction effect between crowding and temperature was analyzed. This information will improve the ability to forecast the occurrence and potential changes in temporal and geographical distribution of this kind of insect under the context of global warming.

## Results

### Crowding effect on survival of nymphs

**Figure 1:**
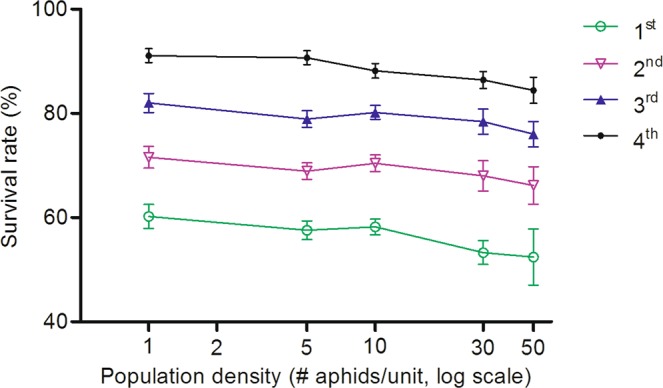
Survivorship (mean ± SE%) of *Rhopalosiphum maidis* at each developmental stage with different population densities under 22 °C.

Crowding induced a rather low (<10%) but constant mortality to all instars by comparing different population density treatments across the lowest to the highest. Accordingly, the survivorship of the aphids reared under five densities was well fitted to the logit model (Table 1). In addition, the survival rate was approximately 10% lower for a younger stage than an elder stage (Fig. 1) under the same population density.

**Table 1:** Values of survivorship at each developmental stage of *Rhopalosiphum maidis* under five densities (Logit Model).

Instar	n	Intercept	Slope	χ^2^	*df*	Heterogeneity
1^st^	450	−0.442 ± 0.082	0.190 ± 0.071	0.919	3	0.306
2^nd^	450	−0.928 ± 0.089	0.129 ± 0.076	0.788	3	0.263
3^rd^	450	−1.512 ± 0.103	0.181 ± 0.088	1.072	3	0.357
4^th^	450	−2.418 ± 0.140	0.397 ± 0.115	1.160	3	0.387

### Crowding induction of alatae

The effect of crowding on alate morphs induction varied among the nymphs. The 1^st^ and 2^nd^ instars were significantly affected by crowding in the production of alate morphs, whereas the 3^rd^ and 4^th^ instars were not significantly affected by crowding (Table 2). In addition, the 1^st^ instar was 2 times more sensitive than the 2^nd^ instar in response to crowding.

**Table 2:** Values of alatae induction at each developmental stage of *Rhopalosiphum maidis* under five densities (Chi-square Contingency Table Analysis).

Instar	*df*	χ^2^	*P*
1^st^	4	24.06	<0.0001
2^nd^	3	9.07	0.0284
3^rd^	3	1.21	0.7504
4^th^	2	0.96	0.6190

**Figure 2:**
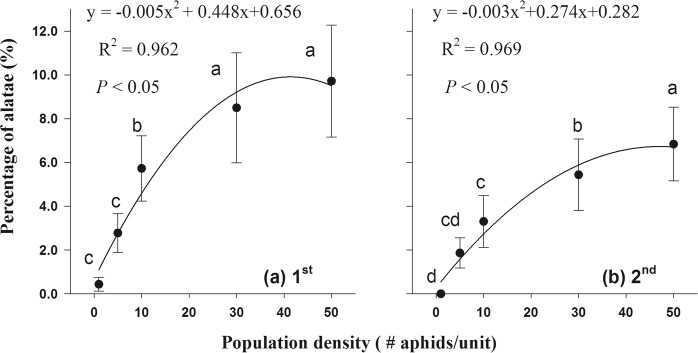
Modulation of population density on alate morphs production of *Rhopalosiphum maidis*: (**a**) Crowding treatment at 1^st^ instar; (**b**) Crowding treatment at 2^nd^ instar. The curved lines in (**a**) and (**b**) were generated based on the regression model using the data shown in the scatterplot in each graph. Different letters indicate significant differences (*P* < 0.05) by using Tukey’s multiple range tests.

For the 1^st^ and 2^nd^ instars, the correlation between alate morph proportion (y_a_ and y_b_) and five empirical population densities was well fitted with second-order polynomial models (Fig. 2a,b), respectively: y_a_ = 0.656 + 0.448x − 0.005x^2^, adjusted R^2^ = 0.962; *P* < 0.05; y_b_ = 0.282 + 0.274x − 0.003x^2^, and adjusted R^2^ = 0.969; *P* < 0.05.

### Maternal effects

**Figure 3:**
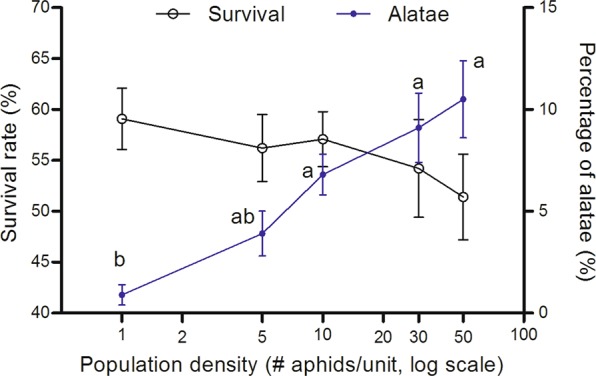
Maternal crowding effect on offspring survival and alatae of *Rhopalosiphum maidis*. Different letters indicate significant differences (*P* < 0.05) by using Tukey’s multiple range tests.

Differences in aphid density in the maternal environment did not influence their survival rates (*F*_4,45_ = 0.61, *P* = 0.659), but impacted their offspring polyphenism (*F*_4,45_ = 6.29, *P* < 0.001) when aphid adults were crowded with five population densities. A strong wing polyphenic response of the first generation was observed. Under the population density of 50, the alatae rate of first generation was approximately 10.5%, which was significantly higher than population density of 1 and 5, which was 0.9% and 3.7%, respectively (Fig. 3).

### Effects of transient high temperatures on survival and alate development

**Figure 4:**
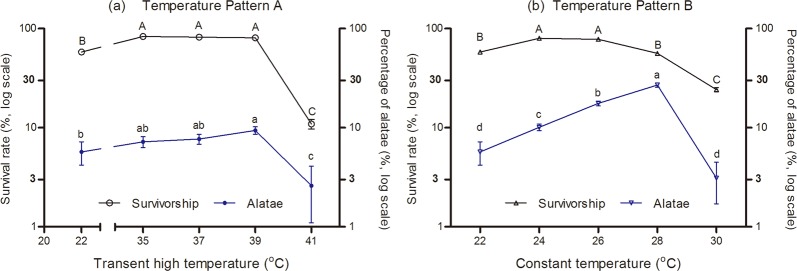
Survivorship and alatae induction (mean ± SE%) of *Rhopalosiphum maidis* at two temperature patterns: (**a**) Temperature pattern A; (**b**) Temperature pattern B. Different capital or lowercase letters represent significant differences (*P* < 0.05) by using Tukey’s multiple range tests.

Temperature pattern A (*F*_4, 220_ = 433.59, *P* < 0.001) and temperature pattern B (*F*_4, 220_ = 227.28, *P* < 0.001) had a significant effect on the survival rate of CLA. The survival rate was significantly increased when the aphids were reared under temperature pattern A, i.e., constant temperature of 22 °C with a transient (2 h) exposure to a high temperature in the range of 35 to 39 °C during mid-photophase (Fig. 4a), whereas 41 °C was detrimental to the aphids, which significantly decreased their survival. In temperature pattern B, the survival rates were significantly high at constant temperatures between 22–26 °C with a transient (2 h) exposure to high temperature of 39 °C during mid-photophase (Fig. 4b). High temperatures (28–30 °C) significantly affected the survivorship of CLA.

Temperature patterns A (*F*_4, 220_ = 9.49, *P* < 0.001) and B (*F*_4, 220_ = 65.53, *P* < 0.001) also resulted in significant differences in the induction of alatae. When nymphs were reared under pattern A, the percentage of alatae generated was increased from 7% to 9% as the transient high temperature increased from 35 to 39 °C. Although there were no significant differences in the induction of alatae among three temperature settings, the proportion of alatae at 22/39 °C was significantly higher than control that at 22 °C (Fig. 4a). When nymphs were reared under the temperature pattern B, the percentages of alatae were significantly different among these settings. In general, as the temperature increased from 24 to 28 °C, the percentage of alatae was increased from 10% to 27%. However, when temperature was as high as 30 °C, the percentage of alatae declined to 3%, while the survival was the lowest (Fig. 4b).

### The interaction effects between crowding and temperature

Crowding and temperature significantly affected the proportion of aphid survival and alatae (Table 3). The temperature was found to have a larger influence than density on both aphid survival and alatae emergence. As the density and temperature accelerated, the proportion of survivors was dramatically decreased, while aphid alatae the rate was significantly increased. The proportion of alatae was increased by up to 41% at 28/39 °C when density was 50 nymphs per tube, while the survival rate was as low as 43% (Table 4). However, no significant interaction effect was found on survival rates and alatae emergence.

**Table 3:** Summarized models of the effects of density^1^ and temperature^2^ on the survival and alatae of *Rhopalosiphum maidis* using General Linear Model (GLM).

Variable	Source	*df*	*F*	*P*
Survival (%)	Density	1	10.22	0.0017
	Temperature	2	96.27	0.0001
	Density _*_ Temperature	2	1.46	0.2359
Alatae (%)	Density	1	16.32	0.0001
	Temperature	2	57.89	0.0001
	Density _*_ Temperature	2	1.56	0.2144

^1^Density: 10 and 50 aphids/unit;

^2^Temperature pattern: 24/39 °C, 26/39 °C and 28/39 °C.

**Table 4:** The percentage of survival and alatae (mean ± SE%) of *Rhopalosiphum maidis* under different temperature and density combinations.

Temperature	Survival (%)		Alatae (%)	
	10 nymphs/unit	50 nymphs/unit	10 nymphs/unit	50 nymphs/unit
24/39 °C	79.9 ± 1.5Aa	77.1 ± 4.4 Aa	10.1 ± 0.8 Aa	13.6 ± 0.9 Aa
26/39 °C	78.2 ± 1.4 Aa	72.2 ± 2.9 Aa	17.6 ± 1.0 Ab	21.7 ± 1.4 Ab
28/39 °C	56.4 ± 1.6 Ab	43.8 ± 2.8 Bb	26.9 ± 1.4 Ac	41.6 ± 1.2 Bc

Within a row, different lowercase letters indicate significant differences (*P* < 0.05) by using Tukey’s multiple range tests;

Within a column, different uppercase letters indicate significant differences (*P* < 0.05) by using Tukey’s multiple range tests.

## Discussion

There are numerous environmental factors that lead to the production of insect alatae forms. Temperature, photoperiod and humidity may all influence the production of winged forms either directly or indirectly through an effect on the host plant^18,20,21,36,37^. These factors were generally considered in continuous conditions, and then, their effects were minimized for shorter duration changes. The host plant is probably another main factor in the development of insect alatae forms^38^ but was also kept fixed by using seedlings of the same age, uniform in size and grown under identical conditions. In this way, only some of the direct effects could be studied. Our results confirmed that in addition to fixed changes in the photoperiod, relative humidity rate or host plant, the insect crowding condition is essential for winged induction. Moreover, the temperature had a vital catalytic role in this process. This study contributes to significant insight into understanding the proximate factors that regulate alternative morph production and, lays the foundation for adaptation and evolution of insect polyphenism under extreme climate with short time exposure.

Winged morph production has been considered a driver of density regulation in aphids, and in many species, the production of winged individuals is strongly density dependent^15,18,19,21,39^. The production of winged individuals among aphid populations is essential for the aphid life cycle and is possibly the best strategy for dispersal and colonization to more optimal environments^35^. Our results confirmed earlier studies and clearly showed that aphids are responsive to crowding. While *R. maidis aphid* reared in isolation rarely gave rise to alate forms, the increase in insect density induced larger proportion of alatae. The younger stage appeared to be more sensitive to the density effect. We crowded 1^st^, 2^nd^, 3^rd^, and 4^th^ nymphs and wingless adults within parthenogenetic embryos. The first two instars and the progeny under high density were more likely to be alatae. The development of the crowding response for the two latter instars is not obvious at any density compared with other stages. It is also a general phenomenon that aphids produce fewer winged than wingless morphs, as former require more resources^15^. Despite the clear effect of crowding for CLA, the effect size was small in comparison with the pea aphid which produces a much higher proportion of winged morphs in response to crowding^40^.

Climate change will cause rapid modification of environmental conditions and an unpredictable occurrence of extremes^41–43^. Extreme events and the gradual increase in average temperatures affect all biological traits, from physiology to life history, also including polyphenism. Low-temperature (the lowest at 8 °C) adapted *N. ribisnigri* not only performed better in survival and reproduction with the temperature increase and reached its best at 20–24 °C, but also produced more alates^44^. Martay *et al*. (2017) modelled weather variables combined with aphid population abundance of 80 species across 12 sites in Great Britain from 1970–2010 and found that winged aphids increased on average by 0.70% annually, of which 62.7% could be accounted for by climate change^45^. The empirical study of elevated temperature and CO_2_ revealed that higher temperature (22 vs. 26 °C) will lead to shorter generation time and higher r_m_ (the largest intrinsic rate of natural increase) value as well as more alate reduction of CLA under global warming in combination with elevated CO_2_, which could result in an increase in population growth and spread, i.e., enhancing risk of serious damage to crops by CLA^46^. Under constant temperature conditions, the optimum temperature for development, survival and reproduction of CLA is at 30 °C, but it is unfavorable to adults at 35 °C^47^. In our study, it could favor the survivorship and alate morphs induction of CLA being reared under constant temperature of 22 °C with a transient exposure (2 h) to high temperature (35 to 39 °C) during mid-photophase. In addition, alate morphs induction was increase along with the rearing temperature rising up in the range of 22 to 28 °C, although the optimum temperature for survival was in the range of 22 to 26 °C. Our results demonstrated that not only global warming, but also the frequent extreme heat events would increase the abundance of CLA under the climate change.

Wing polyphenism is essential for the aphid life cycle and, by allowing for migration to fresh food resources, it may contribute to determining the overall fitness of an aphid population. The effects of external environmental conditions on the wing polyphenism of aphids are likely to be independent and may also be mediated by interacting with multiple factors at the same time. It is still unclear how and when these factors start to work. To understand the costs and benefits, it is important to evaluate the intensity of wing polyphenism associated with different habitat quality indicators. We compared the wing polyphenic response of CLA to crowding and high temperature for a short duration. Our results provide an insight into understanding the two fundamental ecological processes, survival and dispersal. First, the aphids must survive, and then, they should be able to move and disperse. In the future, multiple approaches are necessary to analyze the mechanisms that trigger alternative morph determination by integrating functional “omics” to investigate the biochemical pathways. A better physiological understanding of the wing development of aphids will provide novel targets for studying the evolution of polyphenism in relation to environmental conditions such as extreme climate change and population density from species to community levels. Studying higher levels of organization will allow for a better understanding of ecological and evolutionary responses to forecast how climate change could impact biological diversity.

## Material and Methods

### Ethics statement

For aphids collected in the corn field, no specific permits were required. None of the species used in this study are endangered or protected.

### Plant material

Barley, *Hordeum vulgare* L., was sown in a black plastic pot (7.5 cm diameter and 9 cm high), with approximately 30 seedlings per pot. These plants were cultivated in a greenhouse at 22.0 ± 0.5 °C with 16:8 h (L:D) cycle. When plants grew up to two leaves fully expanded, they were then used for aphid rearing.

### Aphid rearing

A corn leaf aphid, *R. maidis*, colony was reared on barley seedlings that were planted every week in a climate-controlled room at 22 ± 0.5 °C, 65% relative humidity, and a photoperiod of 16 L:8 D h. Aphids were maintained for at least three generations at a low population density (five individuals per plant) prior to being used in the experiments.

Based on the mass rearing of CLA, the development durations of 1^st^ and 4^th^ instars were approximately 2.0 days whereas 2^nd^ and 3^rd^ instars required approximately 1.5 days. Therefore, excepting 1^st^ instars that are new nymphs (<6 h old), the 2^nd^, 3^rd^ and 4^th^ instars used for experiments were approximately 2.0, 3.5, and 5.0 days, respectively. Before the start of the experiments, adult CLA were placed into new pots, and 6 h later, the adults were removed, and new born first instars were reared on the barley seedlings. In this way, we could obtain the synchronously developed stage for respective experiments.

### Experimental design

A glass capillary tube (6 mm diameter and 30 mm height) was used as an experimental cell, which was filled in 3 ml of 2% bactoagar mixed with Miracle-Gro (The Scotts Miracle-Gro Company, Marysville, Ohio) as a medium. A piece of barley seedling (5 mm width × 20 mm height), rather than plants, was inserted into the medium. The top of the glass tube was covered with double-deck gauze to avoid aphid escape. Each tube, with a piece of barley seedling, was used as an experimental unit. One to fifty aphids were infested per unit on the barley seedling in the following experiments. All experiments were carried out in the environmental chamber (Heraeus Group, HPS 500, Germany) at 65 ± 5% RH and a photoperiod of 16 L:8 D, and tested aphids were allowed to develop to adults. Finally, the number of survivors and winged morphs was recorded.

### Crowding experiment

The experiments were conducted at 22 °C including four development stages of aphids (1^st^, 2^nd^, 3^rd^, and 4^th^ instars) and five population densities (1, 5, 10, 30, and 50 aphid(s) per experimental unit). We crowded each stage with five population densities. For example, when the population density was 10, 10 nymphs of the same stage were placed into an experimental unit. Twenty-four hours after crowding, these nymphs were individually transferred to their own experimental unit (for a total of 450 nymphs in each treatment). The numbers of survivors and winged morphs was recorded after these nymphs had developed into adults.

### Maternal effects

The newly enclosed adult aphids were carefully collected from rearing condition using a brush and then placed in the experimental unit at densities of 1, 5, 10, 30, and 50 aphid(s). After 24 h of crowding, one adult from each of 5, 10, 30, and 50 density treatments was randomly selected and placed into a new experimental unit to assess its fecundity. The offspring of the adult were individually transferred to a separate unit daily and allowed to develop into an adult. Finally, survival and winged morphs were recorded. There were 10 replicates for each density.

### Temperature experiment

Each experimental unit infested with 10 1^st^ instars (<6 h) was subjected to two patterns of temperature regimes. Each pattern had four temperature settings. In pattern A, the nymphs were reared at 22 °C with a transient (2 h) high temperature of 35, 37, 39 or 41 °C. The transient (2 h) high temperature exposure was set at mid-photophase. In pattern B, the nymphs were reared at 24, 26, 28, or 30 °C with a transient (2 h) high-temperature of 39 °C. The transient (2 h) high temperature exposure was also set at mid-photophase. The constant environment temperature at 22 °C is considered as the control. All experiments were maintained at 65 ± 10% RH, with a 16:8 h light: dark photoperiod. There were 45 replicates for each treatment. The number of survivors and alatae was checked after tested nymphs developed into adults.

### Assessment of interaction between crowding and temperature

The experiment was conducted as 2 factors with a completely randomized design including three temperature levels (rearing temperature/2 h high temperature: 24/39 °C, 26/39 °C, 28/39 °C) and two density levels (10 and 50 1^st^ instars per unit). There were 45 replicates for each treatment. The number of survivors and alatae was recorded after the 1^st^ instars developed into adults.

### Statistical analysis

The proportion of survival and winged aphids between different treatments was subjected to an analysis of variance by using the general linear model with SAS procedure PROC GLM (SAS V9.2). Before analysis, all data were checked for normality and homogeneity of variances using the Kolmogorov-Smirnov and Levene’s test. The percentage data were normalized using the transformation y = arsine (x/100)^1/2^. Treatment means were compared using Tukey’s multiple range tests to determine significance at a 95% confidence level. The logit model was used to fit the survivorship of the nymphs under five densities. A Chi-square contingency table was used to analyse the crowding effect on alate morphs induction among nymphs. Correlation analysis was used to analyse the relationship between population density and percentage of winged nymphs.
